# Supplementary material for: Lesions of the Head Direction Cell System Increase Hippocampal Place Field Repetition
Source: Curr Biol. 2017 Sep 11;27(17):2706–2712.e2. doi: 10.1016/j.cub.2017.07.071 (PMC5607353; doi:10.1016/j.cub.2017.07.071)
Supplement: Document S1. Figure S1 [file mmc1.pdf]

Current Biology, Volume *27*

Supplemental Information

Lesions of the Head Direction Cell System Increase Hippocampal Place Field Repetition

Bruce Harland, Roddy M. Grieves, David Bett, Rachael Stentiford, Emma R. Wood, and Paul A. Dudchenko

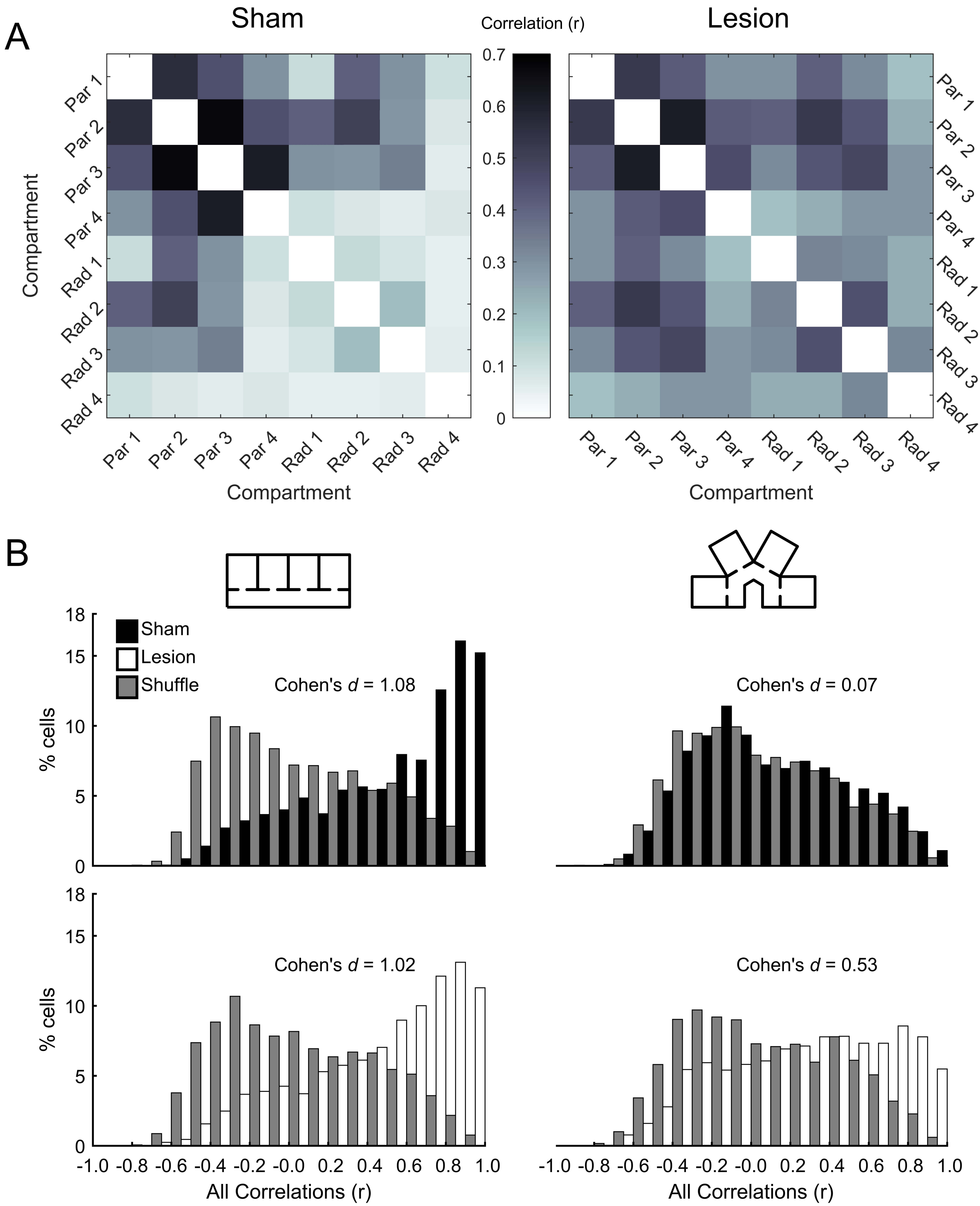

A
Sham
Lesion
Correlation (r)
0.7
0.6
0.5
0.4
0.3
0.2
0.1
0
Par 1
Par 2
Par 3
Par 4
Rad 1
Rad 2
Rad 3
Rad 4
Compartment
B
Sham
Lesion
Shuffle
Cohen's d = 1.08
Cohen's d = 0.07
Cohen's d = 1.02
Cohen's d = 0.53
% cells
18
15
10
5
0
-1.0 -0.8 -0.6 -0.4 -0.2 -0.0 0.2 0.4 0.6 0.8 1.0
All Correlations (r)

Figure S1. Distributions of place field firing map correlations for parallel and radial compartments. Related to Figure 2.

(A) Firing rate map correlations between the four individual compartments in the parallel (Par) and radial (Rad) configurations. For the Sham group, the correlations between compartment maps tended to be high between pairs of parallel compartments (Par 1 vs. Par2; Par 2 vs. Par3, etc.), and low between pairs of radial compartments (e.g., Rad1 vs. Rad2). For the LMN-lesion group, in contrast, the difference in correlations between the parallel and radial compartments was less marked.

(B) Comparison of the observed firing rate map correlations and the shuffled distributions. Across parallel compartments, the correlation of firing rate maps for both Sham and LMN-lesion rats were markedly higher (Sham: mean: .50, SEM: .01; LMN: mean: .47, SEM: .01) than the shuffled distribution of correlations (Sham: mean: .07, SEM: .01; LMN: mean: .07, SEM: .01). In contrast, the distribution of observed radial correlations for the Sham group (mean: .09, SEM: .01) were similar to the shuffled distribution (mean: .07, SEM: .01). The distribution of observed LMN radial correlations (mean: .29, SEM: .01) also differed from the shuffled data (mean: .08, SEM: .01), but not to the extent seen in the parallel compartment correlations.
