# Supplementary material for: Lesions of the Head Direction Cell System Increase Hippocampal Place Field Repetition
Source: Curr Biol. 2017 Sep 11;27(17):2706–2712.e2. doi: 10.1016/j.cub.2017.07.071 (PMC5607353; doi:10.1016/j.cub.2017.07.071)
Supplement: Document S2. Article plus Supplemental Information [file mmc2.pdf]

Report

Current Biology

Lesions of the Head Direction Cell System Increase Hippocampal Place Field Repetition

Graphical Abstract

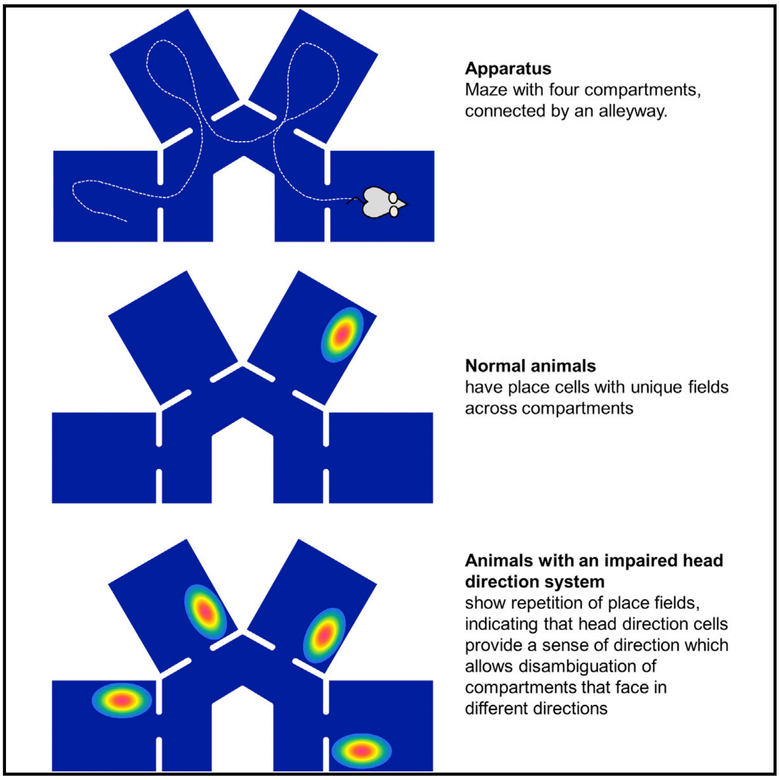

Authors

Bruce Harland, Roddy M. Grieves, David Bett, Rachael Stentiford, Emma R. Wood, Paul A. Dudchenko

Correspondence

p.a.dudchenko@stir.ac.uk

In Brief

Harland et al. show that lesions of the lateral mammillary nuclei, essential nodes in the head direction circuit, increase place field repetition across maze compartments facing different directions. This suggests that place cells and head direction cells work together to allow disambiguation of similar local environments.

Highlights

- Place fields repeat across parallel maze compartments, but not radial compartments
- Lateral mammillary nuclei lesions increase field repetition in radial compartments
- Head direction cells may drive place fields

Harland et al., 2017, Current Biology *27*, 2706–2712
September 11, 2017 © 2017 The Authors. Published by Elsevier Ltd.
http://dx.doi.org/10.1016/j.cub.2017.07.071

Current Biology
Report

Lesions of the Head Direction Cell System Increase Hippocampal Place Field Repetition

Bruce Harland,[1,2,4] Roddy M. Grieves,[1,2,3,4] David Bett,[1,2] Rachael Stentiford,[2] Emma R. Wood,[2,5,6] and Paul A. Dudchenko[1,2,5,6,7,*]
[1]Faculty of Natural Sciences, University of Stirling, Stirling FK9 4LA, UK
[2]Centre for Cognitive and Neural Systems, Edinburgh Medical School: Biomedical Sciences, University of Edinburgh, 1 George Square, Edinburgh EH8 9JZ, UK
[3]University College London, Institute of Behavioural Neuroscience, Department of Experimental Psychology, London, UK
[4]These authors contributed equally
[5]These authors contributed equally
[6]Senior author
[7]Lead Contact
*Correspondence: p.a.dudchenko@stir.ac.uk
http://dx.doi.org/10.1016/j.cub.2017.07.071

SUMMARY

A central tenet of systems neuroscience is that the mammalian hippocampus provides a cognitive map of the environment. This view is supported by the finding of place cells, neurons whose firing is tuned to specific locations in an animal's environment, within this brain region. Recent work, however, has shown that these cells repeat their firing fields across visually identical maze compartments [1, 2]. This repetition is not observed if these compartments face different directions, suggesting that place cells use a directional input to differentiate otherwise similar local environments [3, 4]. A clear candidate for this input is the head direction cell system. To test this, we disrupted the head direction cell system by lesioning the lateral mammillary nuclei and then recorded place cells as rats explored multiple, connected compartments, oriented in the same or in different directions. As shown previously, we found that place cells in control animals exhibited repeated fields in compartments arranged in parallel, but not in compartments facing different directions. In contrast, the place cells of animals with lesions of the head direction cell system exhibited repeating fields in both conditions. Thus, directional information provided by the head direction cell system appears essential for the angular disambiguation by place cells of visually identical compartments.

RESULTS

Hippocampal place cells show repetition of fields in similar, parallel maze compartments but fail to show repetition when the same compartments are oriented in different directions [4]. To test whether the head direction system underlies this sensitivity to direction, we compared place cell repetition in control rats and in rats with damage to the head direction cell system. 15 Lister Hooded rats (6 with lesions of the lateral mammillary nuclei (LMN) of >60% volume; 6 with sham lesions; 3 were excluded because of insufficient lesions) were screened for cells in a cylindrical environment and, upon identification of place cells, recorded in parallel and radial multi-compartment environments (Figure 1A). The environments were placed within a 2-m-diameter black curtained enclosure, which did not contain any explicit polarizing landmarks. Each recording session consisted of an 8-min cylinder session, an 18-min session exploring the parallel compartments, an 18-min session exploring the radial compartments, and then a final 8-min cylinder session. The two cylinder recordings permitted an assessment of basic place cell properties. The order of the parallel and radial recordings was counterbalanced across sessions. Across all animals, data were obtained in 131 recording sessions (mean = 10.9 [±6.7] sessions/animal; range: 2–22).

Place Fields in Both the Sham and LMN-Lesioned Groups Show Repetition in Parallel Compartments, but Only the LMN-Lesioned Animals Show Repetition in Radially Arranged Compartments

For the sham animals, repetition of place fields was observed when the maze compartments were arranged in parallel, but place field repetition was less prominent when the same compartments faced different directions (Figure 1B, left). These results are consistent with the findings in parallel compartments by Spiers et al. [2] and with the parallel and radial compartment recordings of Grieves et al. [4]. In contrast, for rats with lesions of the LMN, place fields repeated across maze compartments both when the compartments faced the same direction and when they faced different directions (Figure 1B, right).

To quantify this repetition, for every place cell, the rate maps for each compartment within a given configuration (parallel or radial) were correlated with one another. The average of these correlations was calculated for each cell and then for all cells for a given animal. As different animals contributed different numbers of cells, this was the most conservative assessment of differences between groups. In this analysis, a place field that repeats across compartments will show higher correlation values across compartments compared to a place field that is unique to a single compartment. We observed that the amount of place field repetition (that is, the mean correlation for firing rates maps across

This is an open access article under the CC BY license (http://creativecommons.org/licenses/by/4.0/).

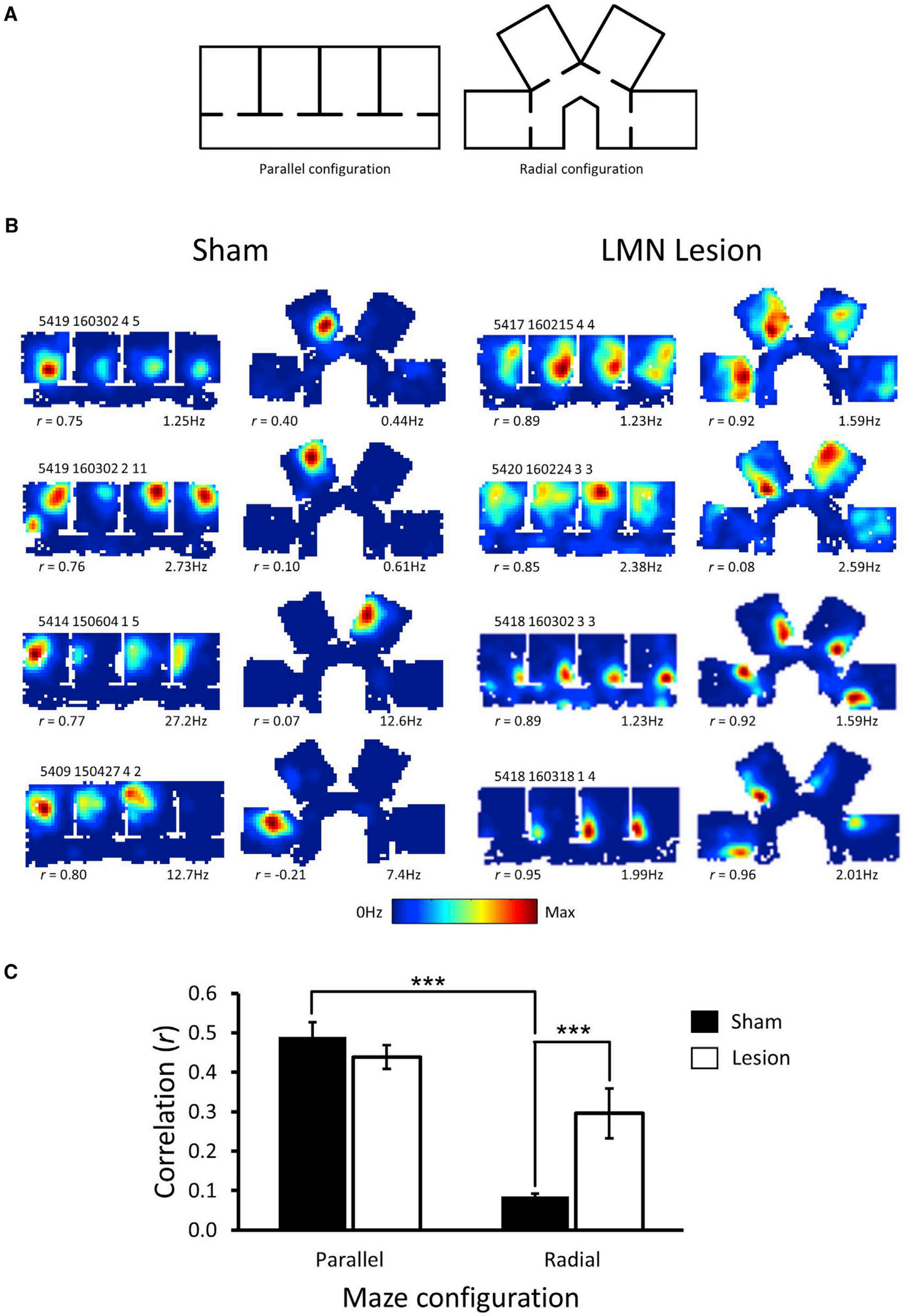

Figure 1. Hippocampal Place Field Repetition in Rats with Sham Lesions and Rats with Lesions of the LMN

(A) Place cells were recorded in an apparatus with four identical compartments. The compartments were arranged in parallel for one recording session and then radially for a second session. The order of these sessions was randomized.

(legend continued on next page)

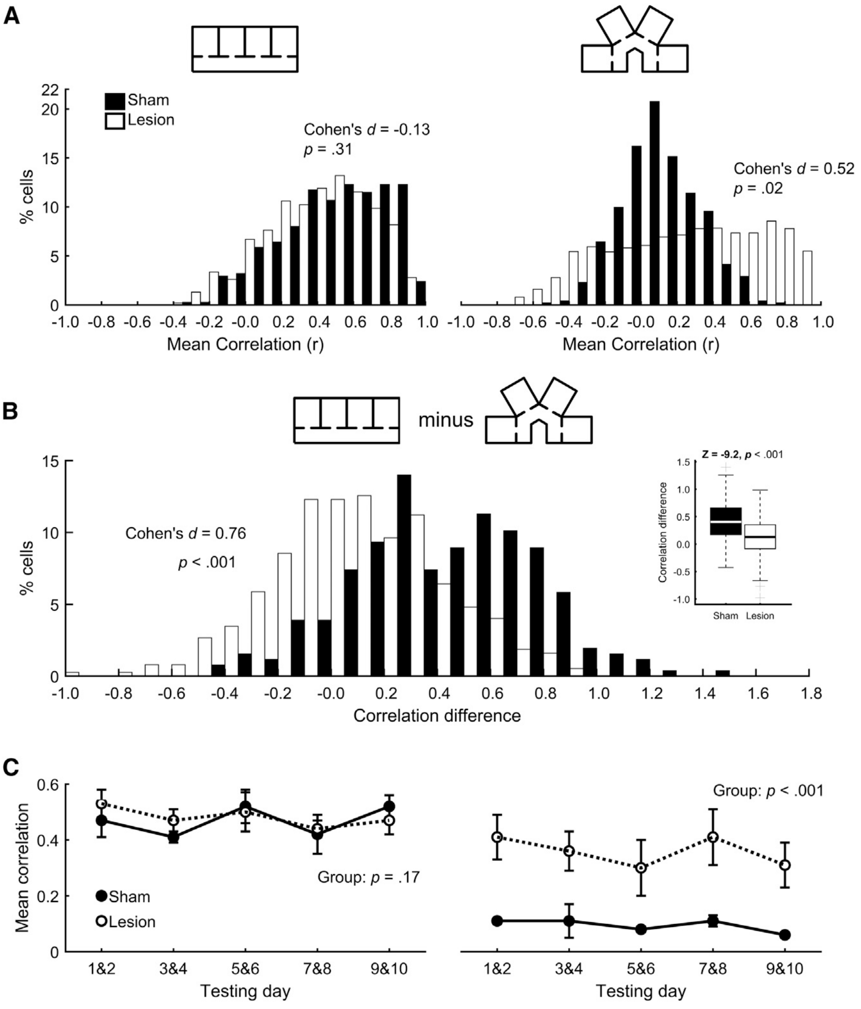

Figure 2. Differences in Correlations between Parallel and Radial Compartments for the Sham and LMN-Lesion Groups

(A) In the parallel compartments, the distribution of place field map correlations is similar for the Sham and Lesion groups (left). However, in the radial compartments, the distribution of correlations for the Lesion group is shifted to the right of the distribution for the Sham group (right). This indicates that rats with LMN lesion showed greater place field repetition in the radial compartments compared with the Sham group (see also Figure S1).

(B) The difference between parallel and radial compartment firing rate map correlations was significantly greater for the Sham group compared with the LMN-lesion group. Overall, this difference was significant (inset plot).

(C) Across the first 10 testing days, place field repetition, as indexed by the correlation of the place field firing rate maps across compartments, was high and stable in the parallel compartments for both the Sham and the LMN-lesion groups (left). In contrast, in the radial compartments, the correlations were low for the Sham group and higher for the LMN-lesion group (right). Error bars indicate SEM.

compartments) in the parallel and radial compartments depended upon whether the animals were in the Sham or LMN-lesioned groups (mixed-design ANOVA; maze × group interaction: $F(1,10) = 13.60$, $p < 0.005$; $\eta^2 = 0.58$). For the Sham group, parallel maze compartment correlations were much higher than those in the radial maze (paired samples t test: $t(5) = 11.56$; $p < 0.001$). This difference was not observed in the LMN-lesion group ($t(5) = 2.34$; $p = 0.066$; see also Figure S1A). Between-group comparisons reveal that parallel maze correlations were equally high in both the Lesion and Sham group (Figure 1C; independent samples t test: $t(10) = 1.06$; $p = 0.31$). However, correlations between radial maze compartment maps were much higher in the Lesion group (independent samples t test [equal variances not assumed]: $t(5.2) = 3.30$; $p = 0.021$). Thus, removal of the LMN diminished the capacity of place cells to distinguish maze compartments that face different directions.

The Distribution of Correlations in the Parallel and Radial Compartments Indicates that LMN-Lesioned Animals Showed More Place Field Repetition in the Radial Compartments Compared with Sham-Lesioned Animals

The distributions of correlations between compartments for those cells that were active in both the parallel and radial sessions are shown in Figure 2A. In the parallel compartments (left plot), place cells from both the Sham and the LMN-lesion groups tended to show repeated fields, and thus, the majority of correlations between compartments were high ($r \geq 0.5$; 65% for the Sham group; 63% for the LMN-lesion group). In contrast, place cells from the Sham group show less repetition of fields in the radial compartments, and thus, the majority of their correlations (82%) here were low ($r \leq 0.5$). In contrast, a substantial fraction (44%) of cells in the LMN-lesioned animals showed high ($r \geq 0.5$) correlations between compartments in the radial configuration.

To test whether the observed place field activity differed from randomly located fields, the observed correlations were compared with shuffled correlation distributions for place cells that fired in both parallel and radial compartments (Figure S1B). Due to the large sample sizes in these comparisons, effect size

(B) In animals with sham lesions (left), place field repetition was observed when the four compartments were arranged in parallel, but not when they were arranged in different directions. In contrast, in animals with lesions of the LMN (right), place field repetition was observed in both parallel and radial compartments. Warmer colors indicate higher rates of firing.

(C) Across the parallel compartments, place field maps were highly correlated for both the Sham and the LMN-lesion groups. However, in the radial compartments, the LMN-lesioned group showed significantly higher correlations across compartments compared to the Sham group. Bars indicate means across animals, and error bars indicate SEM.

(Cohen's *d*) is a more appropriate measure of the difference between the observed correlations and the shuffled distributions (see [4]). Similar, large effect sizes were found for the Sham parallel ($d = 1.08$) and LMN-lesion parallel ($d = 1.02$) observed versus shuffled correlations. This suggests a comparable level of place field repetition in the parallel maze. In contrast, the effect size for the LMN radial correlations ($d = 0.53$) was at an intermediate level between the effect size for the Sham radial correlations ($d = 0.07$) and those seen in the parallel maze.

The comparable level of place field repetition in the parallel maze for both Sham and LMN-lesion rats allowed us to use "difference metrics" [3] to determine whether these groups exhibited any differences in terms of rate remapping. Sham and LMN-lesion rats exhibited similar levels of rate remapping (independent samples t test: $t(911) = 0.69$; $p = 0.49$) in the parallel maze. Moreover, the distribution of observed difference metrics in the parallel maze for both Sham and LMN-lesion rats was not significantly different from the shuffled distributions of difference metrics (one-sample t tests: Sham: $t(748) = -0.45$, $p = 0.65$; LMN: $t(1074) = -0.48$, $p = 0.63$). This analysis does not preclude the possibility that differences in firing rates across parallel compartments are sufficient to allow disambiguation of these at the level of the hippocampus, though previous analyses suggest this modulation is not strong [2].

To quantify the changes in place fields in the radial and parallel compartments, we subtracted each cell's mean correlation across the radial compartments from that of the same cell's mean correlation in the parallel compartments (for cells that fired in both maze configurations). High, positive values in this difference score indicate that place field repetition was present in the parallel compartments, but not in the radial compartments. Values closer to zero indicate a comparable amount of place cell repetition in both compartment configurations. Overall, the parallel-radial differences in compartment correlations for the Sham group were larger than the differences observed in the LMN-lesioned animals (one-way ANOVA: $F(1, 629) = 101.2$; $p < 0.001$). The distributions of these differences for the two groups are shown in Figure 2B. As is evident in this figure, the distribution of parallel-radial differences for the LMN-lesioned animals is shifted to the left compared with the distribution of these differences in the Sham group.

Place Cells in LMN-Lesioned Animals Showed Repetition between Radial Compartments throughout Testing

This pattern of results described above was robust and consistent across 10 testing sessions. During this time, correlations in the parallel compartments did not differ between the Sham and LMN-lesioned groups and did not change across days (Figure 2C, left plot; group effect: $F(1,36) = 2.01$, $p = 0.17$; day effect: $F(4,36) = 0.47$, $p = 0.76$; group × day interaction: $F(4,36) = 1.22$, $p = 0.32$). In contrast, for the radial compartments, place fields in the LMN-lesioned animals showed consistently more repetition than those of the Sham group (Figure 2C, right plot; group effect: $F(1,36) = 28.8$; $p < 0.001$). This difference was stable across testing sessions (day effect: $F(4,36) = 0.04$, $p = 0.98$; group × day interaction: $F(4,36) = 0.02$, $p = 0.99$).

Lesions of the LMN Diminish Place Field Cohesiveness and Stability

In the cylinder sessions, 697 place cells were recorded in LMN-lesioned animals, and 652 cells were recorded in sham animals. The number of unique cells is likely to be lower, as we did not attempt to distinguish cells across recording sessions (though the electrodes were advanced after each session). To compare groups, we calculated an average value of each place cell measure for each rat. Compared to sham-lesioned animals, place cells in LMN-lesioned animals had lower spatial information (LMN: mean 1.05 b/s, SEM: 0.044; Sham: mean: 1.29 b/s, SEM: 0.041; $F(1,10) = 20.32$, $p < 0.002$; $\eta^2 = 0.67$), higher sparsity (LMN: mean 0.37, SEM: 0.012; Sham: mean: 0.32, SEM: 0.009; $F(1,10) = 16.95$, $p < 0.005$; $\eta^2 = 0.63$), and a lower correlation between cylinder firing rate maps (LMN: mean: 0.347, SEM: 0.047; Sham: mean: 0.620, SEM: 0.047; $F(1,10) = 17.0$, $p < 0.005$; $\eta^2 = 0.63$). However, the firing rates of place cells in LMN-lesioned animals did not differ from those in the sham animals (maximum firing rate: $F(1,10) = 1.75$, $p = 0.15$; overall firing rate: $F(1,10) = 0.81$, $p = 0.39$). This pattern of results is in agreement with an earlier study by Calton et al. [5]. They found that, compared with control rats, those with lesions of the anterior dorsal thalamus or the postsubiculum produced a significant decrease in place cell spatial coherence, shifts in fields between cylinder sessions, and a decrease in spatial coherence (though this last effect did not reach significance). Similarly, Sharp and Koestler [6] found that lesions of the medial and lateral mammillary bodies together produced a significant decrease in place field coherence without an effect on firing rates.

Histology

Bilateral infusions of ibotenic acid produced a range of cell loss within the LMN from 62.7% to 100% with a mean lesion size of 82.7%. Three animals with sub-total lesions (11%, 20.5%, and 33.4%) were excluded from the analyses above, as the lesions were judged to be incomplete. A representative coronal section at the level of the LMN from a lesion and a control animal is presented in Figure 3.

DISCUSSION

Previous work has shown that place cells exhibit a robust disambiguation of identical compartments when they face different directions, but not when they face the same direction [4]. The current experiment tested whether central nodes in the head direction cell network, the LMN, are essential for this discrimination. Our results show that damage to the LMN increases place field sparsity, decreases spatial information, and decreases place cell differentiation of compartments facing different directions. We consider these findings below.

Place Fields Are Driven by Borders and by Directional Inputs

The repetition of place fields in maze compartments of the same shape and orientation suggests that place cells are driven by local boundaries [1–3, 7]. Evidence also indicates that place cells are sensitive to direction. In rats, place cells in dorsal portions of the hippocampus typically fire in only one location on a plus maze or a radial arm maze, despite

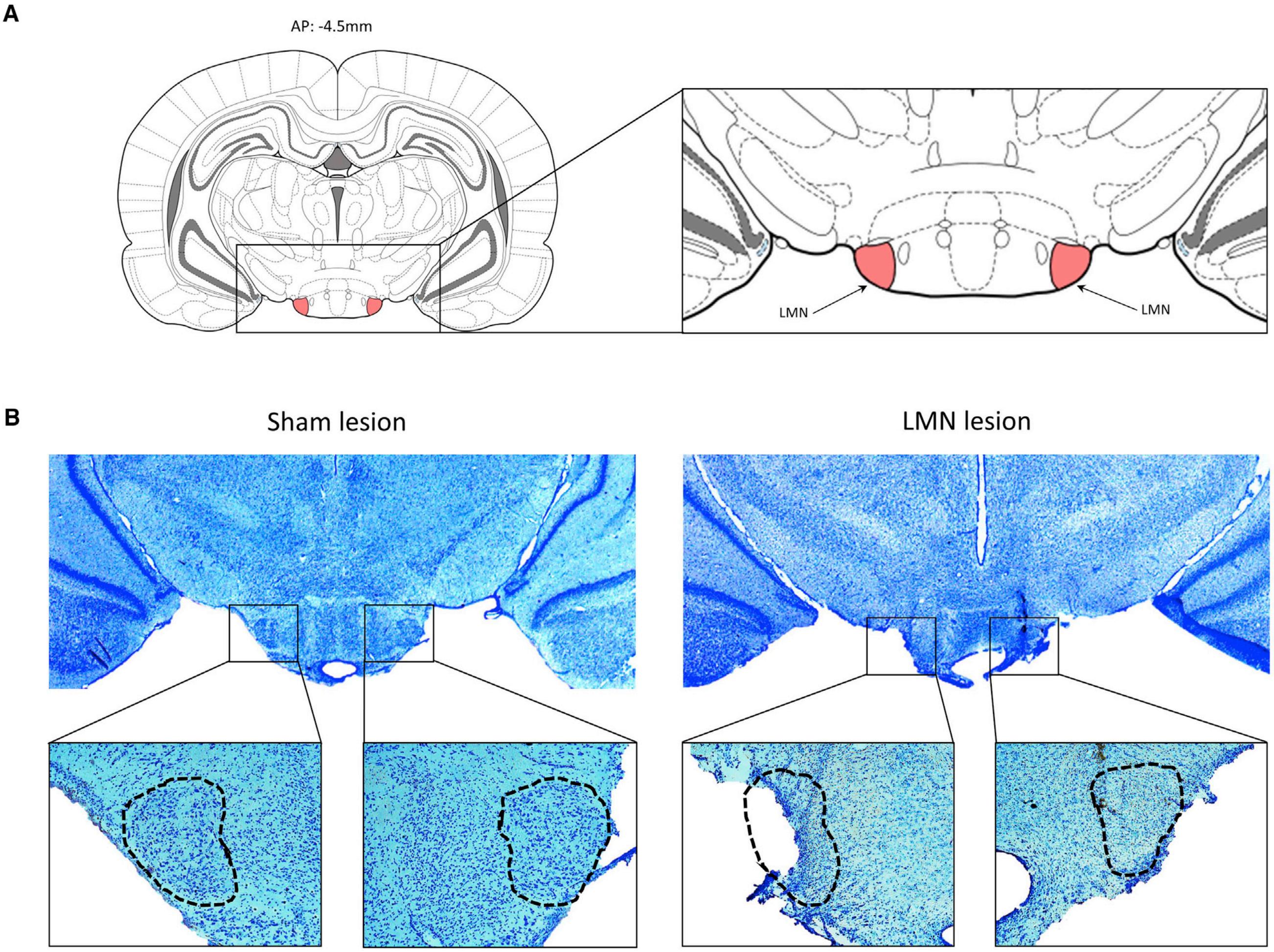

Figure 3. Photomicrograph of LMN in Representative Sections from an Animal in the Sham Group and from the LMN-Lesion Group
The top plot (A) shows the location of the LMN, and the bottom photos (B) are of Nissl-stained sections from the two groups at lower and higher magnifications.

there being four or eight similar maze arms [8–10]. They also fire when the animal traverses a maze segment in one direction, but not the opposite direction [1, 11–13] (but see [14]). The same sensitivity is not observed in the ventral CA3 cells, and cells therein show less theta modulation, and firing on all arms of a radial maze [15].

Grieves et al. [4] provided evidence for a sensitivity to local boundaries and allocentric direction by demonstrating that the place fields of cells in dorsal CA1 repeat across four identical compartments when these are arranged in parallel, but not when they are arranged in different orientations. Thus, when compartments have similar boundaries and the same orientation, place field repetition is observed. However, when compartments have the same boundaries but different orientations, less place field repetition is observed. This suggests that both boundaries and a directional input influence the firing of place cells. Indeed, accumulating evidence indicates that directional signals are present within the hippocampus [16–18]. There is also evidence from brown bats and Egyptian fruit bats that CA1 place cells also show directional tuning [19].

The current results indicate that lesions of the LMN yield an increase in place field repetition in radially arranged compartments. The LMN contains head direction cells [20, 21] and is an essential node in the head direction circuit as lesions of this structure abolish the head direction (HD) signal in the anterior dorsal thalamus (ADN) [21, 22]. Prior work has examined the effects of lesions to either the ADN or the postsubiculum (PoS), both head-direction-cell-containing regions, on place cells [5]. Lesions to each increase place cell directionality in a cylindrical recording environment, and PoS lesions weaken landmark stimulus control over place fields. The rationale for lesioning the LMN in the current study is that it abolishes head direction signals in the ADN and PoS and potentially the directional input to the nucleus reuniens, an additional region recently found to contain head direction cells [23]. Manipulations of the HD circuit upstream of the LMN (in the nucleus prepositus hypoglossi projections to the dorsal tegmental nuclei) cause drift in the head direction signal and inaccuracy in a homing task [24]. The ADN is necessary for normal head direction firing in the postsubiculum [25] and for both head direction and tuned grid cell activity in the medial entorhinal cortex [26]. The diminished sensitivity of

place fields to compartment orientation in LMN-lesioned animals observed in the current study is consistent with a loss of head direction cell input.

The current finding is consistent with the view that the head direction system maintains a global (or at least laboratory room) reference as the animal moves back and forth through different compartments [27, 28]. When the compartments are arranged in parallel, a given head direction cell fires in the same way—for example, north—in each compartment. Combined with an identical set of local boundaries, the walls of the compartments, this stable directional input (provided by multiple head direction cells) would yield similar place fields in each compartment. In contrast, when compartments face different directions, a given head direction cell's firing would be stable in laboratory-based coordinates but would differ relative to each compartment's walls. The combination of directional and local boundary information in this instance would provide a unique input to a given place cell in each compartment, which presumably would give rise to uncorrelated place fields. Removal of the head direction input, as done here, removes the mismatch between global direction and local boundaries. In this case, place cells are driven only by the local boundaries in each compartment, regardless of the compartment's orientation.

Under this scenario, the head direction system is necessary to allow the hippocampus to disambiguate local environments that face different directions. In the parallel compartments, it is not necessary for repetition of place fields, as repetition occurs in both rats with or without LMN lesion. We hypothesize that the head direction cell system provides an invariant representation of direction across compartments, which combines with an input representing boundaries to yield place fields. We have implied that one function of CA1 place cells is disambiguation of similar local environments, but an alternative possibility is that hippocampus functions to generalize across similar environments.

Data that may speak to these alternatives are found in a recent study by Carpenter et al. [28]. They found that grid cell initially represented two identical maze compartments in the same way (i.e., locally). With repeated experience in the maze, however, the grid cell representation spanned both compartments and thus provided a global representation of the apparatus. We did not observe this change across sessions in the current study, though this may reflect differences in duration of the exposures to the compartments and/or the number of compartments to be distinguished. We speculate that, with an experience-based global representation, place cells would disambiguate even parallel compartments. Further, we predict that this disambiguation would not occur, even with extensive experience, in the absence of the head direction cell system, as it is necessary for normal grid cell firing [26].

An unresolved question is whether the impact of LMN (head direction cell) removal on place cells is direct or whether it occurs via other types of spatially tuned neurons. There is evidence that damage to the head direction cell circuit disrupts grid cell spatial tuning [26]. However, accumulating evidence suggests that place cell firing is not dependent on grid cell inputs [29]. Alternatively, disruptions of the head direction circuit may alter the directional sensitivity of boundary vector/border cells, which in turn alters place cell sensitivity to compartment orientation. Assessment of this possibility requires further experimentation.

Some Sensitivity to Compartment Orientation Is Preserved in Animals with LMN Lesions

Though the current results are consistent with a contribution of the head direction cell system to place field firing, the diminished sensitivity to compartment orientation was not complete in all animals. In part, this could reflect variability in LMN-lesion size, as animals with larger lesions tended to show more place field repetition in the radial compartments. It is also possible that the LMN is not the sole source of directional input to the hippocampus. There is evidence that different cells in the entorhinal cortex possess different directional tuning [30], and it could be speculated that this arises from inputs that are not dependent on the integrity of the LMN. However, inactivation of the anterior thalamus abolishes directional firing from the medial entorhinal cortex (MEC) [5], suggesting that the LMN-anterior thalamus directional input is main source of directional inputs to the hippocampus.

Conclusions

The current data suggest that the head direction cell system contributes to the ability of the hippocampus to disambiguate local environments that face different directions. This suggests that angular integration, most likely represented in the activity of head direction cells, underlies global representations of complex space.

STAR★METHODS

Detailed methods are provided in the online version of this paper and include the following:

- KEY RESOURCES TABLE
- CONTACT FOR RESOURCE SHARING
- EXPERIMENTAL MODEL AND SUBJECT DETAILS
- METHOD DETAILS
  - Surgery
  - Electrophysiological recording
  - Histology
- QUANTIFICATION AND STATISTICAL ANALYSIS
  - Place cell repetition analyses
- DATA AVAILABILITY

SUPPLEMENTAL INFORMATION

Supplemental Information includes one figure and can be found with this article online at http://dx.doi.org/10.1016/j.cub.2017.07.071.

AUTHOR CONTRIBUTIONS

Conceptualization, B.H., R.M.G., E.R.W., and P.A.D.; Methodology, B.H., R.M.G., E.R.W., and P.A.D.; Software, R.M.G.; Analyses, B.H., R.M.G., R.S., E.R.W., and P.A.D.; Investigation, B.H., D.B., and R.S.; Writing, B.H., R.M.G., R.S., D.B., E.R.W., and P.A.D.; Funding Acquisition, E.R.W. and P.A.D.

ACKNOWLEDGMENTS

We wish to thank Ms. Jane Tulloch for her assistance with histology. This work was supported by Biotechnology and Biological Sciences Research Council (BBSRC) grant BB/LOOOO40/1, awarded to P.A.D.

Received: May 26, 2017
Revised: July 2, 2017
Accepted: July 31, 2017
Published: August 31, 2017

REFERENCES

1. Derdikman, D., Whitlock, J.R., Tsao, A., Fyhn, M., Hafting, T., Moser, M.-B., and Moser, E.I. (2009). Fragmentation of grid cell maps in a multicompartment environment. Nat. Neurosci. *12*, 1325–1332.
2. Spiers, H.J., Hayman, R.M., Jovalekic, A., Marozzi, E., and Jeffery, K.J. (2015). Place field repetition and purely local remapping in a multicompartment environment. Cereb. Cortex *25*, 10–25.
3. Fuhs, M.C., Vanrhoads, S.R., Casale, A.E., McNaughton, B., and Touretzky, D.S. (2005). Influence of path integration versus environmental orientation on place cell remapping between visually identical environments. J. Neurophysiol. *94*, 2603–2616.
4. Grieves, R.M., Jenkins, B.W., Harland, B.C., Wood, E.R., and Dudchenko, P.A. (2016). Place field repetition and spatial learning in a multicompartment environment. Hippocampus *26*, 118–134.
5. Calton, J.L., Stackman, R.W., Goodridge, J.P., Archey, W.B., Dudchenko, P.A., and Taube, J.S. (2003). Hippocampal place cell instability after lesions of the head direction cell network. J. Neurosci. *23*, 9719–9731.
6. Sharp, P.E., and Koester, K. (2008). Lesions of the mammillary body region severely disrupt the cortical head direction, but not place cell signal. Hippocampus *18*, 766–784.
7. Skaggs, W.E., and McNaughton, B.L. (1998). Spatial firing properties of hippocampal CA1 populations in an environment containing two visually identical regions. J. Neurosci. *18*, 8455–8466.
8. Olton, D.S., Branch, M., and Best, P.J. (1978). Spatial correlates of hippocampal unit activity. Exp. Neurol. *58*, 387–409.
9. O'Keefe, J., and Speakman, A. (1987). Single unit activity in the rat hippocampus during a spatial memory task. Exp. Brain Res. *68*, 1–27.
10. Mizumori, S.J.Y., McNaughton, B.L., Barnes, C.A., and Fox, K.B. (1989). Preserved spatial coding in hippocampal CA1 pyramidal cells during reversible suppression of CA3c output: evidence for pattern completion in hippocampus. J. Neurosci. *9*, 3915–3928.
11. McNaughton, B.L., Barnes, C.A., and O'Keefe, J. (1983). The contributions of position, direction, and velocity to single unit activity in the hippocampus of freely-moving rats. Exp. Brain Res. *52*, 41–49.
12. Muller, R.U., Bostock, E., Taube, J.S., and Kubie, J.L. (1994). On the directional firing properties of hippocampal place cells. J. Neurosci. *14*, 7235–7251.
13. Dombeck, D.A., Harvey, C.D., Tian, L., Looger, L.L., and Tank, D.W. (2010). Functional imaging of hippocampal place cells at cellular resolution during virtual navigation. Nat. Neurosci. *13*, 1433–1440.
14. Battaglia, F.P., Sutherland, G.R., and McNaughton, B.L. (2004). Local sensory cues and place cell directionality: additional evidence of prospective coding in the hippocampus. J. Neurosci. *24*, 4541–4550.
15. Royer, S., Sirota, A., Patel, J., and Buzsáki, G. (2010). Distinct representations and theta dynamics in dorsal and ventral hippocampus. J. Neurosci. *30*, 1777–1787.
16. Leutgeb, S., Ragozzino, K.E., and Mizumori, S.J. (2000). Convergence of head direction and place information in the CA1 region of hippocampus. Neuroscience *100*, 11–19.
17. Acharya, L., Aghajan, Z.M., Vuong, C., Moore, J.J., and Mehta, M.R. (2016). Causal influence of visual cues on hippocampal directional selectivity. Cell *164*, 197–207.
18. Peyrache, A., Roux, L., Schieferstein, N., and Buzsaki, G. (2016). Transformation of head-direction signal into spatial code. bioRxiv. http://dx.doi.org/10.1101/075986.
19. Rubin, A., Yartsev, M.M., and Ulanovsky, N. (2014). Encoding of head direction by hippocampal place cells in bats. J. Neurosci. *34*, 1067–1080.
20. Stackman, R.W., and Taube, J.S. (1998). Firing properties of rat lateral mammillary single units: head direction, head pitch, and angular head velocity. J. Neurosci. *18*, 9020–9037.
21. Blair, H.T., Cho, J., and Sharp, P.E. (1998). Role of the lateral mammillary nucleus in the rat head direction circuit: a combined single unit recording and lesion study. Neuron *21*, 1387–1397.
22. Blair, H.T., Cho, J., and Sharp, P.E. (1999). The anterior thalamic head-direction signal is abolished by bilateral but not unilateral lesions of the lateral mammillary nucleus. J. Neurosci. *19*, 6673–6683.
23. Jankowski, M.M., Islam, M.N., Wright, N.F., Vann, S.D., Erichsen, J.T., Aggleton, J.P., and O'Mara, S.M. (2014). Nucleus reuniens of the thalamus contains head direction cells. eLife *3*, e03075.
24. Butler, W.N., Smith, K.S., van der Meer, M.A.A., and Taube, J.S. (2017). The head-direction signal plays a functional role as a neural compass during navigation. Curr. Biol. *27*, 1259–1267.
25. Goodridge, J.P., and Taube, J.S. (1997). Interaction between the postsubiculum and anterior thalamus in the generation of head direction cell activity. J. Neurosci. *17*, 9315–9330.
26. Winter, S.S., Clark, B.J., and Taube, J.S. (2015). Spatial navigation. Disruption of the head direction cell network impairs the parahippocampal grid cell signal. Science *347*, 870–874.
27. Dudchenko, P.A., and Taube, J.S. (1997). Correlation between head direction cell activity and spatial behavior on a radial arm maze. Behav. Neurosci. *111*, 3–19.
28. Carpenter, F., Manson, D., Jeffery, K., Burgess, N., and Barry, C. (2015). Grid cells form a global representation of connected environments. Curr. Biol. *25*, 1176–1182.
29. Bush, D., Barry, C., and Burgess, N. (2014). What do grid cells contribute to place cell firing? Trends Neurosci. *37*, 136–145.
30. Giocomo, L.M., Stensola, T., Bonnevie, T., Van Cauter, T., Moser, M.-B., and Moser, E.I. (2014). Topography of head direction cells in medial entorhinal cortex. Curr. Biol. *24*, 252–262.
31. Harland, B., Wood, E.R., and Dudchenko, P.A. (2015). The head direction cell system and behavior: the effects of lesions to the lateral mammillary bodies on spatial memory in a novel landmark task and in the water maze. Behav. Neurosci. *129*, 709–719.
32. Kadir, S.N., Goodman, D.F., and Harris, K.D. (2014). High-dimensional cluster analysis with the masked EM algorithm. Neural Comput. *26*, 2379–2394.
33. Hazan, L., Zugaro, M., and Buzsáki, G. (2006). Klusters, NeuroScope, NDManager: a free software suite for neurophysiological data processing and visualization. J. Neurosci. Methods *155*, 207–216.

STAR★METHODS

KEY RESOURCES TABLE

REAGENT or RESOURCE	SOURCE	IDENTIFIER
Chemicals, Peptides, and Recombinant Proteins		
Ibotenic acid	Tocris Bioscience	Cat. #0285
Deposited Data		
University of StirIng DataSTORRE	https://datastorre.stir.ac.uk/	Harland Data
Experimental Models: Organisms/Strains		
Lister Hooded Rats	Charles Rivers Laboratories, UK	N/A
Software and Algorithms		
IBM SPSS Statistics	IBM	v.21
KlustaKwik	http://klustakwik.sourceforge.net/	v.1.5
Klusters	http://neurosuite.sourceforge.net/	N/A
Image Pro Plus	Media Cybernetics, USA	v. 6.2
ImageJ	Wayne Rasband, NIH, USA	1.46j
Other		
Axona Recording System	Axona, St. Albans, U.K.	DacqUSB

CONTACT FOR RESOURCE SHARING

Further information and requests for resources should be directed to and will be fulfilled by the Lead Contact, Paul Dudchenko (p.a.dudchenko@stir.ac.uk).

EXPERIMENTAL MODEL AND SUBJECT DETAILS

Fifteen adult, male Lister Hooded rats (Charles Rivers Laboratories, UK) weighing 250-350 at the start of the experiment served as subjects. All procedures complied with the UK Animals (Scientific Procedures) Act (1986) and the European Communities Council Directive of November 24, 1986 (86/609/EEC). All animal experiments were carried out in compliance with protocols approved by the University of Edinburgh Animal Welfare and Ethical Review Board (AWERB), and under a UK Home Office Project License.

METHOD DETAILS

Surgery

Rats were anaesthetised with isoflurane (Abbott, UK), and either received bilateral ibotenic acid infusions in the lateral mammillary nuclei (via a 1-μL Hamilton syringe angled at 10 degrees to AP – 4.5 mm, ML ± 1.0 mm, DV −9.2 mm) or a sham surgery (where the dura was pierced, but no infusions were made); see [31] for additional details. In the same surgery, all animals were also implanted with tetrode microdrives directed toward the dorsal CA1 layer of the hippocampus (−3.48 mm AP from bregma, ± 2.4 mm ML from the midline, −1.8 mm DV from dura surface). For additional details see [4].

Electrophysiological recording

Single units were assessed with an Axona USB recording system (Axona, St. Albans, UK). Upon identification of a place cell, a recording sequence was conducted comprising an initial cylinder session (eight minutes), a session in either the parallel or the radial configurations of the multicompartment apparatus (18 min), a second session of the same duration in the alternate configuration and a final cylinder session of eight minutes. Between the parallel and radial sessions, the maze was washed down, and the compartments were switched with one another. After each recording day, all tetrodes were advanced. The apparatus for these recordings were centered within a circular, black, curtained enclosure, with a white curtain creating a false ceiling. No directionally polarizing cues were provided within this enclosure (aside from the multi-compartment apparatus themselves). The parallel and radial compartments were comprised of 35 × 40 × 30 cm (width x length x height) wooden boxes, and the compartments were connected with alleyways that were 20 cm wide and 30 cm high (see [4]).

Spike data were analyzed with custom MATLAB scripts. Complex-spike cells were identified on the basis of energy, first principal component, peak amplitude, peak time and waveform width using the Klustakwik spike sorting algorithms [32]. Clusters were manually assessed and refined using the manual cluster cutting program Klusters [33].

Histology

At the completion of the experiment, rats were perfused with 4% formalin, and 32 um coronal brain sections were taken from the region of the electrode track and from the region of the lateral mammillary nuclei. Slices were stained with Nissl stain and then coverslipped. Images of the LMN were obtained with a microscope (Leica DMRB, Germany) using 2.5x and 5.0x objectives, a QICAM camera (QImaging, Canada) and Image Pro Plus software (version 6.2; Media Cybernetics, USA). For both the Sham and the LMN-lesioned groups, the size of the LMN was quantified by outlining its extent with ImageJ 1.46h (Wayne Rasband, NIH, USA) software. This produced a measurement of the area within the outline. An average LMN size was calculated for the animals in the Sham group, and any residual LMN tissue in the lesioned animals was expressed as a percentage of this average.

QUANTIFICATION AND STATISTICAL ANALYSIS

Statistical comparisons were conducted with the IBM SPSS Statistics (v.21) package, and are reported in the text of the Results. Statistically significant differences were those with a probability of less than 5% ($p < 0.05$)

Place cell repetition analyses

The extent of place cell repetition in the parallel and radial compartments was quantified using similar methods to Grieves et al. [4]. Briefly, firing rate maps were generated for each of the four compartments within a given maze configuration. Pearson correlations were then calculated for compartment pairs in which the mean firing rate in one or both compartments of the pair exceeded 1 Hz. Before conducting correlations, the radial configuration compartment rate maps were rotated so as to align them with their longest axis vertical and the doorway positioned to the bottom for each cell. The mean of the correlations between compartments was calculated for each environment. To generate shuffled correlation distributions we repeated the above process on shuffled compartment rate maps, where the contributing neuron was shuffled but compartment identity was maintained.

The above analysis is sensitive to changes in spatial firing but is insensitive to changes in firing rate. For this reason we also repeated the above analyses using the 'difference metric' reported by Fuhs et al. [3] instead of the Pearson *r* correlation.

We first zero-normalized the compartment rate maps used above by subtracting their respective mean firing rates. We then calculated the difference metric as:

$$D = \frac{\sum_x |f_1(x) - f_2(x)|}{\sum_x |f_1(x)| + |f_2(x)|}$$

where x ranges over map locations, and f_1(x) and f_2(x) are the two compartment firing rate maps. We calculated this for every possible compartment pair (i.e., 1 v 2, 1 v 3, 1 v 4, 2 v 3, 2 v 4 and 3 v 4) for every cell. This metric is bounded between 0 and 1, where 0 indicates identical fields and 1 indicates fields that are maximally different. This metric is still sensitive to the location of firing fields; if two fields exhibit a similar firing rate but are not spatially aligned the outcome will be close to 1. For this reason the metric is not well suited to comparing compartment maps in the radial maze, where we expect spatial remapping between compartments. However, in the parallel maze, where compartments are represented similarly, this metric indicates the extent to which maps vary in terms of firing rate. For this analysis we also generated a corresponding shuffled distribution, in the same way as described for the correlation analysis.

DATA AVAILABILITY

All cluster-cut recording files will be made available in the University of Stirling's DataSTORRE (https://datastorre.stir.ac.uk/).

Current Biology, Volume *27*

Supplemental Information

Lesions of the Head Direction Cell System

Increase Hippocampal Place Field Repetition

Bruce Harland, Roddy M. Grieves, David Bett, Rachael Stentiford, Emma R. Wood, and Paul A. Dudchenko

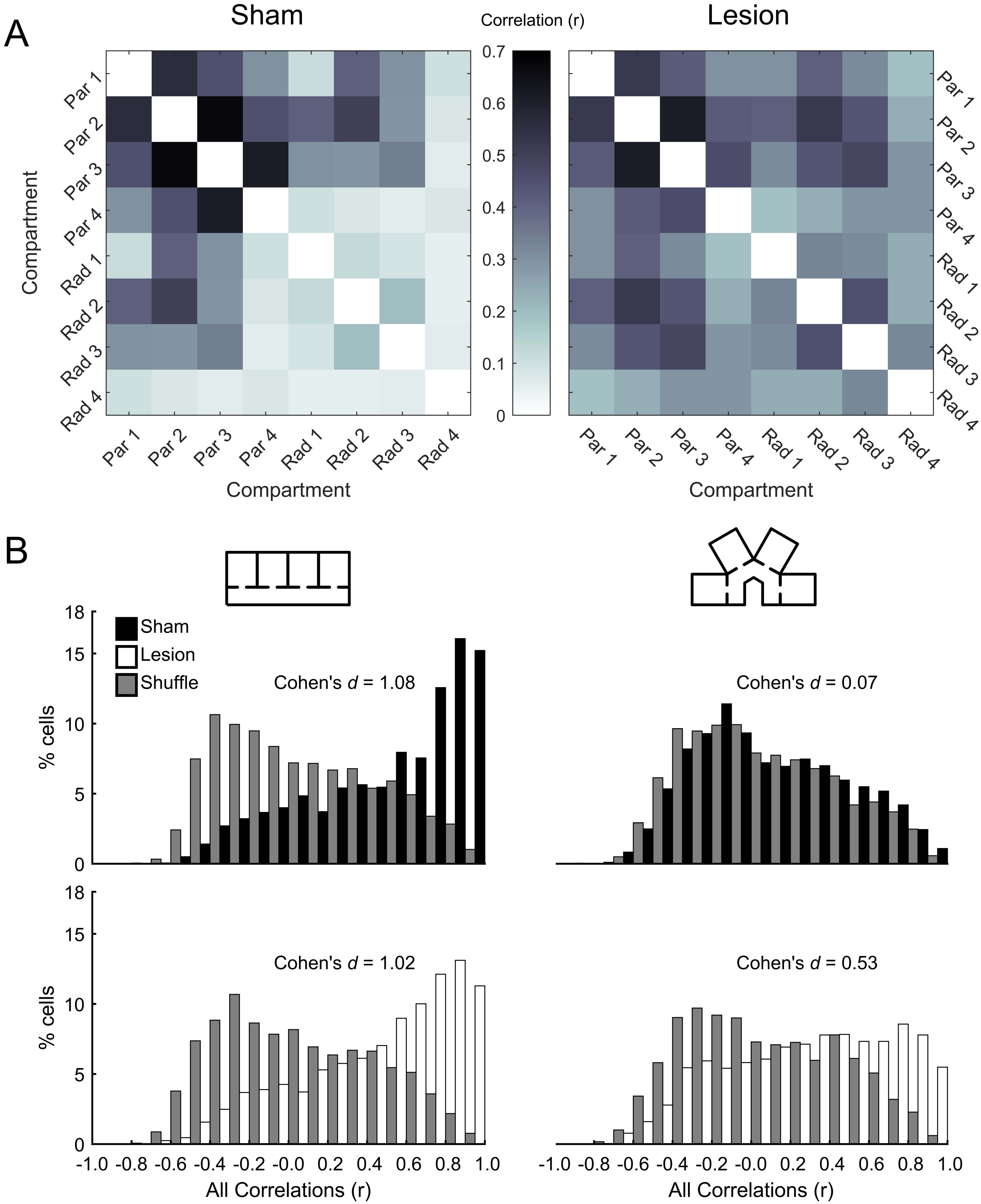

A
Sham
Lesion
Correlation (r)
0.7
0.6
0.5
0.4
0.3
0.2
0.1
0
Par 1
Par 2
Par 3
Par 4
Rad 1
Rad 2
Rad 3
Rad 4
Compartment
B
Sham
Lesion
Shuffle
Cohen's d = 1.08
Cohen's d = 0.07
Cohen's d = 1.02
Cohen's d = 0.53
% cells
18
15
10
5
0
-1.0 -0.8 -0.6 -0.4 -0.2 -0.0 0.2 0.4 0.6 0.8 1.0
All Correlations (r)

Figure S1. Distributions of place field firing map correlations for parallel and radial compartments. Related to Figure 2.

(A) Firing rate map correlations between the four individual compartments in the parallel (Par) and radial (Rad) configurations. For the Sham group, the correlations between compartment maps tended to be high between pairs of parallel compartments (Par 1 vs. Par2; Par 2 vs. Par3, etc.), and low between pairs of radial compartments (e.g., Rad1 vs. Rad2). For the LMN-lesion group, in contrast, the difference in correlations between the parallel and radial compartments was less marked.

(B) Comparison of the observed firing rate map correlations and the shuffled distributions. Across parallel compartments, the correlation of firing rate maps for both Sham and LMN-lesion rats were markedly higher (Sham: mean: .50, SEM: .01; LMN: mean: .47, SEM: .01) than the shuffled distribution of correlations (Sham: mean: .07, SEM: .01; LMN: mean: .07, SEM: .01). In contrast, the distribution of observed radial correlations for the Sham group (mean: .09, SEM: .01) were similar to the shuffled distribution (mean: .07, SEM: .01). The distribution of observed LMN radial correlations (mean: .29, SEM: .01) also differed from the shuffled data (mean: .08, SEM: .01), but not to the extent seen in the parallel compartment correlations.
